# Optimizing Odor-Baited Trap Methods for Collecting Mosquitoes during the Malaria Season in The Gambia

**DOI:** 10.1371/journal.pone.0008167

**Published:** 2009-12-04

**Authors:** Musa Jawara, Renate C. Smallegange, David Jeffries, Davis C. Nwakanma, Taiwo Samson Awolola, Bart G. J. Knols, Willem Takken, David J. Conway

**Affiliations:** 1 Medical Research Council Laboratories, Fajara, Banjul, The Gambia; 2 Laboratory of Entomology, Wageningen University, Wageningen, The Netherlands; Université Pierre et Marie Curie, France

## Abstract

**Background:**

Baited traps are potential tools for removal or surveillance of disease vectors. To optimize the use of counter-flow traps baited with human odor (nylon socks that had been worn for a single day) to capture wild mosquitoes in the Gambia, investigations were conducted at a field experimental site.

**Methodology/Principal Findings:**

Experiments employing Latin square design were conducted with a set of six huts to investigate the effects of the following on overnight mosquito trap catches: (1) placement of traps indoors or immediately outdoors, CO_2_ supply, and presence of a human subject in the hut; (2) trap height for collecting mosquitoes immediately outdoors; (3) height and distance from hut; (4) interaction between multiple traps around a single hut and entry of mosquitoes into huts. A total of 106,600 adult mosquitoes (9.1% *Anopheles gambiae s.l.,* 4.0% other *Anopheles* species) were collected over 42 nights. The high numbers of *An. gambiae s.l*. and other mosquitoes collected by odor-baited traps required CO_2_ but were largely independent of the presence of a person sleeping in the hut or of trap placement indoors or outdoors. For outdoor collection that is considered less intrusive, traps opening 15 cm above the floor of the hut veranda were more highly effective than traps at other heights or further from the hut. There was no significant evidence of saturation or competition by the traps, with multiple traps around a hut each collecting almost as many mosquitoes as single traps and no effect on the numbers of mosquitoes entering the huts.

**Conclusions/Significance:**

The outdoor trapping protocol is convenient to compare attractiveness of different odors or synthetic chemicals to malaria vectors and other wild mosquitoes. The finding that such traps are reliably attractive in the presence or absence of a human volunteer encourages their potential development as standardised surveillance tools.

## Introduction

Host odours play a major role in attracting blood-seeking nocturnal female mosquitoes [1]. The major African malaria vectors of the *Anopheles (An.) gambiae* complex are anthropophilic, with human sweat and skin residues being highly attractive [2], [3], and several candidate chemical component attractants have been identified [4]–[8]. Such knowledge may help develop mosquito traps that can reduce the contacts between vectors and humans, or as a surveillance tool. Initial studies have shown that MM-X ® counter-flow traps are useful to test the attractiveness of natural human skin emanations and synthetic compounds and the repellent effect of plant materials, under enclosed semi-field and open field conditions [9]–[11]. Traps baited with a nylon sock worn by a human subject for 12 hours or more can attract considerable numbers of anopheline mosquitoes under enclosed semi-field conditions [9], [11].

Experimental huts are ideal for studying attractiveness or repellence of odours to vector mosquitoes, before community-based studies are undertaken. For example, it is necessary to determine the best placement of traps in or around dwellings by first using experimental huts. The present study therefore employs a highly attractive human odour bait, in the form of worn socks, to determine optimal use and placement of traps in relation to huts with sleeping volunteers. Four sets of experiments were conducted to determine: (1) if traps are best placed indoors or outdoors, with or without CO_2_, and if a person sleeping inside the hut affects the luring of mosquitoes towards the traps; (2) the best height for collecting mosquitoes immediately outdoors; (3) efficiency of traps at different heights and distances away from the hut; (4) the possibility of competition among outdoor traps and their influence on numbers of mosquitoes entering huts.

Notably, the human odour-baited MM-X trap was proven to be an effective tool to attract large numbers of anopheline and other mosquitoes in and around huts, independently of the presence of human sleepers. The results define a trapping protocol that is suitable for comparing candidate chemical attractants and evaluating their potential use in vector surveillance.

## Materials and Methods

### Ethics statement

This study including the protocols for mosquito trapping and schedule of volunteers sleeping in experimental huts was reviewed and approved by the Gambia Government/MRC Joint Ethics Committee. The mostly illiterate volunteers gave informed consent verbally, as witnessed and documented by the study team. The Ethics Committee approved the consent procedure in this low-risk research project, recognising that the familiarity with and support of the entomology studies at Walikunda by these volunteer men from a neighbouring village is fully testified.

### Study area and experimental site

Walikunda is a small fishing village of ∼50 inhabitants, located on the south bank of the River Gambia in the Central River Region of The Gambia (13° 34' N, 14° 5' W), ∼180 km from the Atlantic coast, within the Sudan savannah belt of West Africa. Apart from the adjacent river on the north east, it is surrounded by rice fields which are rain fed and irrigated. Previous behavioural studies on wild *An. gambiae s.l.* populations have been conducted here in the field [10], [12] and with experimental huts [13]. In Walikunda, two members of the *An. gambiae* complex are common (*An. arabiensis* and *An. gambiae s.s.* M form), whereas the two other taxa that are present elsewhere in The Gambia (*An. melas* and *An. gambiae s.s.* S form) have not been detected locally [14].

**Figure 1:**
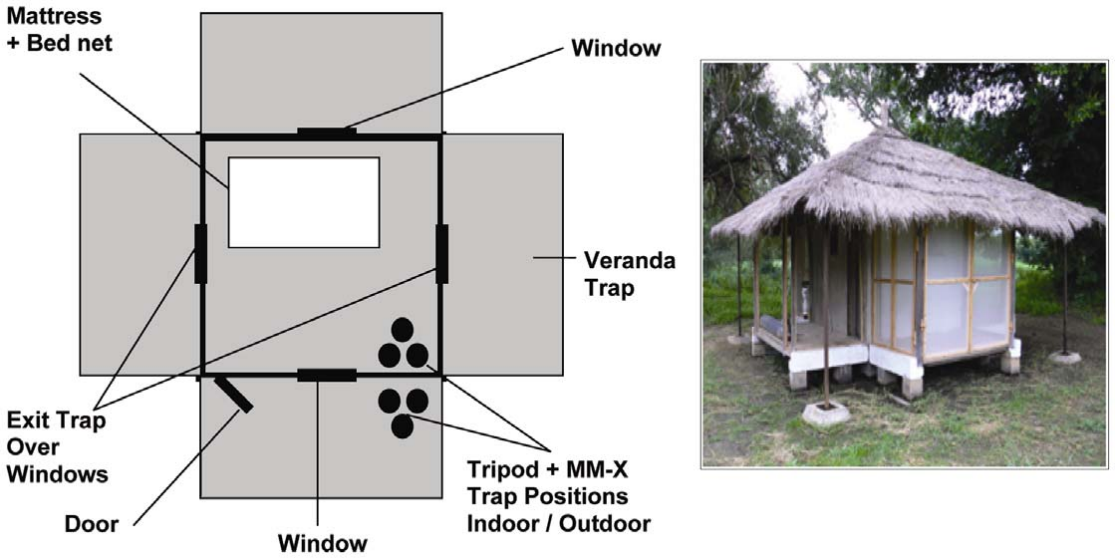
Schematic diagram and photograph of an experimental hut. The diagram shows positions of indoor and outdoor traps, sleeping mattress, doors, verandas, windows (2 facing the open fields opposite each other), and exit traps (2 mounted at the windows enclosed in the veranda traps opposite each other). Half of the six experimental huts had their doors on the left edge and half on the right edge.

At the MRC site in Walikunda, there are six experimental huts (Figure 1) purpose built of a traditional square design with mud walls plastered with cement, plywood ceiling, thatched roof, open eaves, a window in each wall and a door facing north east towards the River Gambia. The main rice fields are situated on the south west side, and the huts are arranged 11 meters apart from each other in a straight line. These huts are designed to measure the number of mosquitoes entering each room at night, allowing for those mosquitoes that entered and subsequently left during the night. Two opposite sides of the huts have window exit traps and screened verandas to capture mosquitoes leaving via the window or eaves. To prevent ants entering the buildings, the huts are raised off the ground on concrete pillars surrounded by small water-filled moats. They have been used for studying mosquito behaviour and measuring the efficacy of different interventions for ∼20 years [13], [15]. Over the experimental period (the annual peak malaria transmission season from August to November 2007) the mean outdoor temperature at the site was 28.3°C, and mean relative humidity was 78.5%. Inside the huts the mean temperature was 28.1°C, and mean relative humidity was 78.7%.

### Experimental hut procedures

Prior to the commencement of each experiment, the experimental huts were cleaned and clean bed nets and bed sheets were placed on the mattresses. Gas cylinders containing CO_2_ (Banjul Oxygen) and tripod stands used for suspending the MM-X traps were also put in place (Figure 1). The traps were placed close to the wall with the door (facing north east) next to the window, either inside or outside for experiments 1 and outside for experiments 2, 3 and 4 (CO_2_ tubing was directed through the window when the trap was set indoors). In experiment 3, traps were positioned at the back (facing South West) at predetermined distances away from the hut, towards the direction of the rice fields. In the evenings at 6.00 pm the windows were slightly opened to leave a gap of about 10 cm, simulating a poorly fitting window as observed in village huts, and they remained in this position until 6.30 am, while the doors remained closed.

Six adult men (ages 17–55) were recruited as volunteers to sleep in the huts. These men have previously all being involved as sleepers in experimental hut studies at Walikunda. The sleepers were randomly allocated to the huts for each experimental series, and each one slept in the same hut for the duration of the series. At the beginning of each experimental series the sleepers were given new shoes to wear, locally purchased and made of canvas or synthetic leather. At the start of the day in the mornings at 7.00 am, they were given a pair of nylon socks (HEMA, The Netherlands) to wear for a period of ∼12 hours daily (7.00 am–8.30 pm), and these were used as bait in the traps. When they took off the socks (e.g. during farming activities and during prayer times) they were asked to note and report this, and instructions were given to minimise interference with the socks at these times. The worn sock in each trap was from the volunteer assigned to sleep in the corresponding hut, and were generally used as immediately worn, except during experiment 4 for which socks had been stored frozen at -20°C to build up sufficient material. Sleepers retired to bed in their allocated huts at 9.00 pm and came out at 7.00 am. They were given anti malarial chemoprophylaxis on a weekly basis.

### Mosquito trapping devices

The Mosquito Magnet Model X (MM-X)® trap (former American Biophysics, USA) was used to assess mosquito density during the experiments. The traps were all new, not previously used and not modified as in Qui et al. [10] except for one trap used within experiment 4 which had previously been modified but was readjusted to the original design. The standard octenol cartridge holders were removed in all the traps. Each MM-X trap was positioned inside or outside the same hut for every night of collection depending on the experimental design running at the time. 100% CO_2_ was delivered from cylinders supplied by Banjul Oxygen through connections to the MM-X traps using silicone tubing (Rubber bv, The Netherlands, 7 m, 7×10 mm) and FLOWSET1 (ABC) tubing. Incorporated into the ABC tubings are a 10 µm line filter and a control orifice (0.007 inches) which when coupled to a regulated gas supply at 15PSI, releases 500 ml/min of CO_2_. The nightly trapping was started at 9 pm each evening when sleepers went to bed, socks were put in traps, CO_2_ flow was started and connections to 12V batteries completed to begin the trapping.

A Centre for Disease Control miniature light trap (CDC trap) was placed in one of the village houses (the house of the Alkalo, village head) in Walikunda to monitor mosquito population density and composition approximately 50 metres away from the experimental huts throughout the period.

### Mosquito collection and identification

At 7.00 am every morning the MM-X traps were stopped by inserting a plug to prevent mosquitoes from escaping, and disconnecting the positive plug to the batteries and switching off the gas regulators. The windows were first closed at 6.30 am and the exit traps blocked with a piece of clothing material to prevent mosquitoes that entered from exiting. As a large proportion of mosquitoes remain alive and active within the MM-X traps, after collection the traps were put in a freezer at −20°C for 2 hours to kill all the mosquitoes before being emptied for identification. By means of a sucking tube / aspirator the verandas and rooms were visually searched for live mosquitoes and these were collected in labelled cups. A 10 minute search was conducted for each hut (room and veranda traps). The exit traps were also emptied into a labelled cup and all the mosquitoes were placed in the freezer at −20°C for ∼2 hours to ensure they had died before being examined for morphological identification. Mosquitoes collected each night were morphologically identified and counted. The *An. gambiae s.l.* females were individually placed in wells of 96-well plates and kept in silica gel, so that a random sample could be later tested for individual species and *An. gambiae s.s.* M and S molecular form identification by PCR-RFLP assay [16]. *Culex* and *Mansonia* mosquitoes were not identified to species, but were counted at genus level.

### Experimental design and data analysis

The experiments were designed as Latin squares which allow for blocking in two directions, in this case by hut and night. Randomisations were based on 6×6 designs and balanced to control for any carry-over effects of treatments. The Latin squares were combined into a Latin rectangle of 6 huts ×12 nights for those experiments that were conducted for 12 nights. Trap counts were analysed using an analysis of variance (Genstat v10) approach and if necessary the counts were square root transformed. Treatments were compared using F-tests and p-values are uncorrected for multiplicity. Inference was corroborated using a negative binomial model fitted in Stata v10. The experimental data was stored in a Microsoft Access database and the figures were drawn using Matlab v7.

## Results

### Overall mosquito species composition

Overall, 106,600 adult mosquitoes were collected for the 4 series of experiments carried out between August and November 2007 (Table 1). *An. gambiae s.l.* constitute 9.1% of this sum, *An. pharoensis* 2.3%, *An. zeimanni* 1.4%, other anophelines 0.3%, *Culex* species 5.3%, *Aedes* 0.4%, and *Mansonia* species 81.2%. Of these total numbers of adult mosquitoes collected, 21.3% were from Experiment 1, 26.5% from Experiment 2, 17.5% from Experiment 3 and 34.7% from Experiment 4. The mosquito species composition was similar among 4,214 female mosquitoes collected in CDC light traps from a single house in Walikunda village on 21 nights during this period (Table S1). Out of the 9690 morphologically identified *An. gambiae s.l* overall, a random sample of 1194 (12.3%) were PCR-RFLP typed to resolve species and molecular forms, of which 20.6% were *An. arabiensis* and 79.4% were *An. gambiae s.s.* M form, proportions broadly similar to those obtained in household collections from the surrounding area in the two previous years [14].

**Table 1:** Total number of female mosquito species collected from MM-X traps and huts for each experimental series (Experiments 1, 2, and 4 were each conducted over12 nights and Experiment 3 over 6 nights).

	Experiment 1		Experiment 2		Experiment 3		Experiment 4	
MOSQUITO SPECIES COLLECTED	Total MM-X	Total Hut	Total MM-X	Total Hut	Total MM-X	Total Hut	Total MM-X	Total Hut
*An. pharoensis*	764	36	888	61	172	5	555	6
*An. zeimani*	120	15	153	47	466	8	673	5
*An. wellcomei*	0	0	0	0	1	0	21	1
*An. squamosus*	7	2	2	1	0	0	0	0
*An. funestus*	1	2	7	1	5	0	42	0
*An. rufipes*	0	0	5	0	22	1	209	4
*An. nili*	0	0	2	3	0	0	0	0
*Culex* spp.	999	640	1797	373	645	70	1090	37
*Mansonia* spp	12389	3679	15995	5516	15068	1353	29416	3107
*Aedes* spp.	156	36	111	36	18	2	56	8
Total female mosquitoes	16298	6386	20515	7767	16940	1735	33409	3550

### Experiment 1: Testing placement of traps and effect of CO_2_ and sleeper

**Figure 2:**
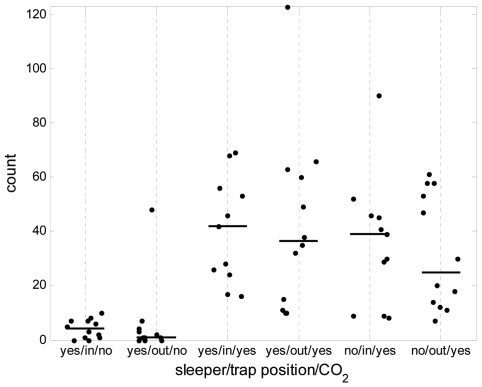
Malaria vector data from Experiment 1. Numbers of female *An. gambiae s.l.* mosquitoes collected in human odour-baited MM-X traps (count per trap) in an experiment investigating the presence or absence of a sleeper in the hut (yes or no), effect of trap position (indoor or outdoor), use of CO_2_ in the trap (yes or no). The x-axis shows each treatment group as a combination of these variables. Horizontal bars show the median count per trap for each treatment group.

The first experiment was to determine whether traps are better placed indoors or outdoors, whether it is influential whether a man sleeps inside the room or not, and whether CO_2_ increases the collections. This was achievable using a double Latin square design with six huts over 12 nights. MM-X traps baited with worn socks were suspended with their lowest part 15 cm above the floor of either the veranda (outside) or the room (inside) of the huts. High numbers of mosquitoes were collected, as with subsequent experiments (Table 1). The results for *An gambiae s.l.* are shown in Figure 2. Traps with CO_2_ gave significantly higher numbers than traps without CO_2_ (P<0.001). The effect of trap position (indoors or outdoors) and the presence or absence of a sleeper did not have any significant effect on the number of *An. gambiae s.l*. mosquitoes collected (P = 0.54 and 0.10 respectively). For the other mosquito genera present in very large numbers (*Culex* and *Mansonia* spp.), outdoor traps collected more than indoor traps (P<0.001, Figure S1), and the use of CO_2_ resulted in higher numbers caught than without CO_2_ (P<0.001). For the total room collections (veranda, room and exit trap combined), significantly less mosquitoes (whether *An. gambiae s.l*., *Mansonia*, or *Culex* spp.) were collected when there was no sleeper in the hut, although this did not significantly influence the numbers of mosquitoes in the MM-X traps (Figure 2 and Figure S1).

On the basis of these results, it was decided to further evaluate the use of the MM-X traps baited with worn socks together with CO_2_ bait (as this was necessary for attracting large numbers of mosquitoes), placed outdoors (as this is less intrusive for human subjects), and in the presence of a sleeper in the hut (as this more closely simulates a community environment).

### Experiments 2 and 3: Optimal height of traps and distance from huts

The next objective was to determine the height and distance away from the hut at which human odour-baited traps are most effective at collecting mosquitoes outdoors. First, using a double Latin square design over 12 nights, traps were set at different heights on the veranda, with trap opening at 15, 30, 50, 100 and 150 cm above the floor (the maximum height that could be investigated was 150 cm as this was underneath the veranda ceiling and parallel to the opening of the eaves). There was a significant non-linear trend with trap height (P<0.001), highest counts of *An. gambiae s.l.* being obtained at the lowest (15 cm) and highest (150 cm) trap heights but no significant difference between these (P = 0.98), whereas for *Culex* and *Mansonia* there were highest numbers trapped at 150 cm (Figure S2). Omnibus tests between all combinations of trap placement showed no significant effects on the room counts (veranda, exit and room totals), so it was decided to further evaluate the efficiency of traps at the heights of 15 cm and 150 cm, placed at a range of distances away from the experimental huts.

**Figure 3:**
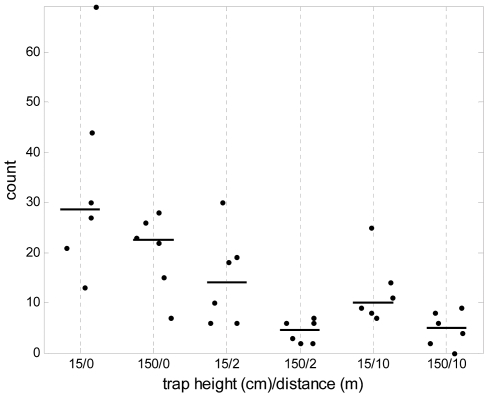
Malaria vector data from Experiment 3. Numbers of female *An. gambiae s.l.* mosquitoes collected in CO_2_ and human odour-baited MM-X traps in an experiment of different trap heights (height in cm of the bottom of the trap containing the opening) and distances away from the huts (in metres, m). Horizontal bars show the count per trap for each treatment group.

For this next experiment the south western side of each hut (facing the main rice fields in the area) was used for placement of the traps, at two different heights (15 cm and 150 cm) and three different distances (0, 2, and 10 m), and the experiment was run through one Latin square (the set of 6 huts over 6 nights). Female *An. gambiae s.l.* mosquitoes were caught in larger numbers in traps located 0 m away from the huts (immediately outside) compared to those 2 m or 10 m away (P<0.001 for each comparison) (Figure 3). Overall, at the height of 15 cm significantly more *An. gambiae s.l.* were collected than at 150 cm (P = 0.001). As in the previous experiment, traps at both of these heights caught abundant numbers of *Culex* and *Mansonia* (data not shown). Again, there was no significant difference in the numbers of mosquitoes collected within the huts, for the different trap positions. It was concluded that the optimal placement of human odour-baited MM-X traps is at a height of 15 cm immediately outside huts to maximize capture of wild *An. gambiae s.l.* mosquitoes, and this was adopted as a standard protocol for further work.

### Experiment 4: Effect of single or multiple traps around a hut

The final objective was to ascertain whether placing more odour-baited traps around a hut would influence the total number of mosquitoes being collected indoors, and to see whether there was evidence of saturation or competition between adjacent traps. It was considered that traps might either attract mosquitoes away from a human sleeper, or possibly that traps might attract more mosquitoes towards the hut. There were four different interventional treatments with traps placed immediately outside the huts: (1) a single trap on the south west, (2) a single trap on the north east, (3) a trap on each of two sides, south west and north east), and (4) a trap on each of the four sides of the hut. The remaining treatments 5 and 6 were controls to gauge the entry of mosquitoes into huts without odour baited traps.

**Figure 4:**
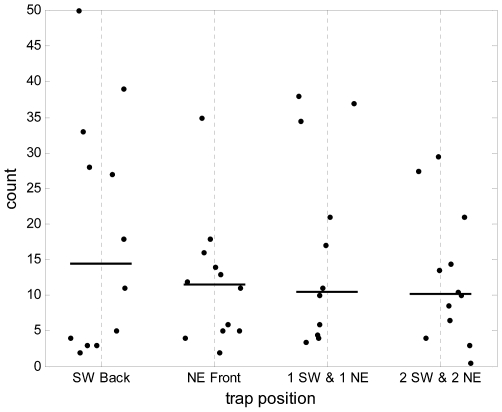
Malaria vector data from Experiment 4. Numbers of female *An. gambiae s.l.* mosquitoes collected per trap in human odour-baited MM-X traps when there are single traps outside huts, either to the south west (SW) or north east (NE), or when there are two or four traps around each hut. For the multiple trap combinations the points plotted represent the mean number of mosquitoes per trap (the total number trapped divided by either two or four as appropriate).

The numbers of *An. gambiae s.l.* collected within each trap did not differ significantly whether there were single or multiple (2 or 4 traps) around each hut ((P>0.1, Figure 4). The numbers of *An. gambiae s.l.* collected within the huts at the end of each night was not significantly affected by the presence of single or multiple traps immediately outside, compared to huts that had no trap outside (Figure S3).

## Discussion

This study shows that MM-X counter-flow traps baited with human odour are effective in catching high numbers of wild *An. gambiae s.l.* females that transmit malaria locally as well as *Culex* and *Mansonia* nuisance mosquitoes. The release of CO_2_ had a major effect in increasing numbers of the mosquitoes caught in the baited MM-X traps, consistent with previous field studies indicating that CO_2_ enhances odour-baited catches [10], [17], [18]. An important finding of this study was that the presence or absence of a sleeper and the position of the trap (outdoor or indoor) did not significantly affect the catch numbers. This indicates that the traps baited with human odour and CO_2_ are robustly attractive even when placed outdoors, and can be used to sample mosquitoes without major confounding from people sleeping nearby. Traps immediately outside the huts caught more *An. gambiae s.l.* females at heights of 15 cm (near ankle height) or 150 cm (adjacent to the eaves) than at intermediate heights, and further testing showed that traps set at 15 cm from the ground had higher catches than traps set at 150 cm at any distance from the huts. For studies on relative attractiveness of different synthetic compounds to mosquitoes, it can be recommended to use traps outdoors at 15 cm height, in close vicinity to huts or houses.

Further work is needed to determine if such traps can be effective in reducing mosquito populations significantly by removal trapping [19], [20]. Here, preliminary experimentation with single and multiple traps around huts indicated that the traps operate largely independently of each other, and that multiple traps allow collection of large numbers of mosquitoes. However, these traps did not cause a decrease in numbers of mosquitoes entering the huts. Therefore, other measures are needed to prevent house entry, including the screening of entry points into houses by netting [21]. Another option could be to use odours that ‘push’ mosquitoes away from human dwellings, following the strategy of push-pull or stimulo-deterrent methods used in agriculture [22]. Repellents, including essential plant oils or synthetic compounds mimicking the odour of repellent plant species [23], [24] could potentially be used by placing them in the eaves of houses.

This study demonstrates effective trapping methods that could be applied to compare different attractants of malaria vectors and nuisance mosquitoes during the period of peak malaria transmission. Odour baited traps clearly offer a promising means to survey vector mosquitoes, as they can be applied in the absence of a human host and allow for standardised simultaneous use of multiple traps. The utility of odour baited traps in comparison with other methods needs to be determined in different settings. An advantage for some research purposes is that most of the mosquitoes remain alive in the traps, enabling investigation of their behaviour, physiology and pathogen biology, but this is less advantageous for routine surveillance as it requires an additional step to kill the mosquitoes. In the West African savannah region, it would be worthwhile evaluating their performance for surveillance during the long dry season when malaria vector mosquito collections by other available methods yield very low numbers [25]. In other regions, it would be relevant to test such traps for sampling malaria vectors that inhabit less accessible outdoor sites such as the forest canopy, for which alternative trapping methods are very demanding [26].

## Supporting Information

Figure S1 Numbers of female mosquitoes trapped in the MM-X traps in Experiment 1. (A) Culex spp. (B) Mansonia spp.
(0.06 MB PPT)

Figure S2 Numbers of female mosquitoes trapped in the MM-X traps in Experiment 2. (A) An. gambiae s.l. (B) Culex spp. (C) Mansonia spp.
(0.06 MB PPT)

Figure S3 Numbers of female *An. gambiae s.l.* mosquitoes collected inside huts (veranda, room, and exit traps combined) that had different numbers of baited MM-X traps (0, 1, 2, or 4) placed north east (NE) or south west (SW) immediately outside each hut in Experiment 4.
(0.02 MB DOC)

Table S1 Female mosquitoes collected from a CDC light trap in a single house in Walikunda village to gauge the species composition and density during the experimental period.
(0.08 MB DOC)
