# Supplementary material for: Optimizing Odor-Baited Trap Methods for Collecting Mosquitoes during the Malaria Season in The Gambia
Source: PLoS One. 2009 Dec 4;4(12):e8167. doi: 10.1371/journal.pone.0008167 (PMC2780730; doi:10.1371/journal.pone.0008167)
Supplement: Figure S2 — Numbers of female mosquitoes trapped in the MM-X traps in Experiment 2. (A) An. gambiae s.l. (B) Culex spp. (C) Mansonia spp. (0.06 MB PPT) [file pone.0008167.s002.ppt]

## Slide 1
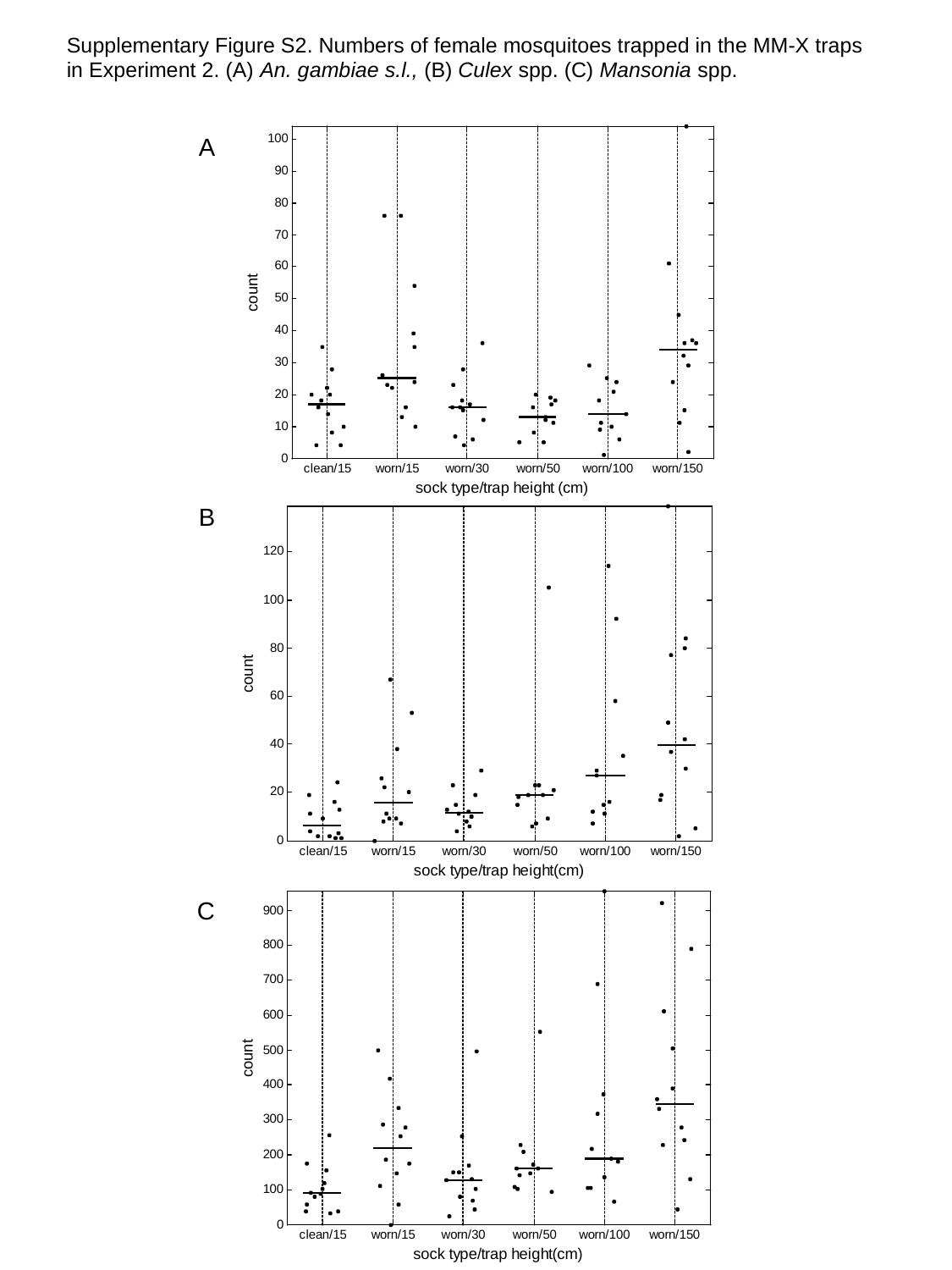

Supplementary Figure S2. Numbers of female mosquitoes trapped in the MM-X traps
in Experiment 2. (A) An. gambiae s.l., (B) Culex spp. (C) Mansonia spp.
A
B
C
