# Supplementary material for: Optimizing Odor-Baited Trap Methods for Collecting Mosquitoes during the Malaria Season in The Gambia
Source: PLoS One. 2009 Dec 4;4(12):e8167. doi: 10.1371/journal.pone.0008167 (PMC2780730; doi:10.1371/journal.pone.0008167)
Supplement: Figure S3 — Numbers of female An. gambiae s.l. mosquitoes collected inside huts (veranda, room, and exit traps combined) that had different numbers of baited MM-X traps (0, 1, 2, or 4) placed north east (NE) or south west (SW) immediately outside each hut in Experiment 4. (0.02 MB DOC) [file pone.0008167.s003.doc]

Supplementary Figure S3. Numbers of female *An. gambiae s.l.* mosquitoes collected inside huts (veranda, room, and exit traps combined) that had different numbers of baited MM-X traps (0, 1, 2, or 4) placed north east (NE) or south west (SW) immediately outside each hut in Experiment 4
