# Supplementary material for: Optimizing Odor-Baited Trap Methods for Collecting Mosquitoes during the Malaria Season in The Gambia
Source: PLoS One. 2009 Dec 4;4(12):e8167. doi: 10.1371/journal.pone.0008167 (PMC2780730; doi:10.1371/journal.pone.0008167)
Supplement: Table S1 — Female mosquitoes collected from a CDC light trap in a single house in Walikunda village to gauge the species composition and density during the experimental period. (0.08 MB DOC) [file pone.0008167.s004.doc]

**Supplementary Table S1.** Female mosquitoes collected from a CDC light trap in a single house in Walikunda village to gauge the species composition and density during the experimental period (between 25/8/07 and 6/10/07).

|  | DATE | | | | | | | | | | | | | | | | | | | | | |
| --- | --- | --- | --- | --- | --- | --- | --- | --- | --- | --- | --- | --- | --- | --- | --- | --- | --- | --- | --- | --- | --- | --- |
| **FEMALE MOSQUITOES COLLECTED** | **25/8/07** | **26/8/07** | **28/8/07** | **29/8/07** | **30/8/07** | **31/8/07** | **1/9/07** | **11/9/07** | **12/9/07** | **13/9/07** | **14/9/07** | **15/9/07** | **16/9/07** | **19/9/07** | **20/9/07** | **21/9/07** | **22/9/07** | **23/9/07** | **24/9/07** | **5/10/07** | **6/10/07** | **Totals** |
| *An.gambiae s.l.* | 41 | 46 | 42 | 27 | 49 | 19 | 48 | 98 | 144 | 11 | 24 | 6 | 11 | 28 | 7 | 17 | 8 | 47 | 44 | 5 | 6 | 728 |
| *An. pharoensis* | 2 | 1 | 0 | 1 | 4 | 2 | 26 | 1 | 2 | 2 | 1 | 0 | 4 | 2 | 0 | 0 | 0 | 4 | 2 | 0 | 0 | 54 |
| *An. zeimani* | 1 | 5 | 0 | 1 | 0 | 0 | 141 | 10 | 11 | 18 | 9 | 7 | 6 | 18 | 21 | 0 | 3 | 1 | 11 | 0 | 0 | 263 |
| *An. welcomei* | 0 | 0 | 0 | 0 | 0 | 0 | 0 | 0 | 0 | 0 | 0 | 0 | 0 | 0 | 0 | 0 | 0 | 0 | 0 | 0 | 0 | 0 |
| *An. squamosus* | 0 | 0 | 0 | 0 | 0 | 0 | 0 | 0 | 0 | 0 | 0 | 0 | 0 | 0 | 0 | 0 | 0 | 0 | 0 | 0 | 0 | 0 |
| *An. funestus* | 0 | 0 | 0 | 0 | 0 | 0 | 0 | 0 | 1 | 1 | 0 | 0 | 0 | 0 | 0 | 0 | 0 | 1 | 0 | 0 | 0 | 3 |
| *An .rufipes* | 0 | 0 | 0 | 0 | 0 | 0 | 0 | 0 | 0 | 0 | 0 | 0 | 0 | 0 | 0 | 0 | 0 | 0 | 0 | 0 | 0 | 0 |
| *An nili* | 0 | 0 | 0 | 0 | 0 | 0 | 0 | 0 | 0 | 0 | 0 | 0 | 0 | 0 | 0 | 0 | 0 | 0 | 3 | 0 | 0 | 3 |
| *Culex* spp. | 11 | 4 | 0 | 4 | 10 | 4 | 18 | 17 | 10 | 10 | 21 | 25 | 0 | 6 | 4 | 8 | 2 | 11 | 4 | 5 | 4 | 178 |
| *Mansonia* spp. | 193 | 46 | 148 | 97 | 189 | 155 | 172 | 223 | 267 | 25 | 120 | 151 | 66 | 150 | 106 | 117 | 143 | 156 | 247 | 133 | 80 | 2984 |
| *Aedes* spp. | 0 | 0 | 0 | 0 | 0 | 0 | 0 | 0 | 0 | 0 | 0 | 0 | 0 | 0 | 0 | 0 | 1 | 0 | 0 | 0 | 0 | 1 |
| Total female mosquitoes | 248 | 102 | 190 | 130 | 252 | 180 | 405 | 349 | 435 | 67 | 175 | 189 | 87 | 204 | 138 | 142 | 157 | 220 | 311 | 143 | 90 | 4214 |
